# Elotuzumab for the treatment of multiple myeloma

**DOI:** 10.1186/s13045-016-0284-z

**Published:** 2016-07-15

**Authors:** Yucai Wang, Larysa Sanchez, David S. Siegel, Michael L. Wang

**Affiliations:** Department of Medicine, Rutgers New Jersey Medical School, Newark, NJ USA; Division of Multiple Myeloma, John Theurer Cancer Center, Hackensack University Medical Center, Hackensack, NJ USA; Department of Lymphoma/Myeloma, Division of Cancer Medicine, The University of Texas M. D. Anderson Cancer Center, 1515 Holcombe Blvd., Unit 429, Houston, TX 77030 USA

## Abstract

Elotuzumab is one of the first two monoclonal antibodies that gained FDA approval for the treatment of multiple myeloma (MM). It targets SLAMF7, which is highly expressed in normal plasma and MM cells as well as natural killer (NK) cells. Elotuzumab demonstrated significant anti-myeloma activity in preclinical studies, and its mechanisms of action include mediating antibody-dependent cell-mediated cytotoxicity, enhancing cytotoxicity of NK cells, and inhibiting MM cell interaction with bone marrow stromal cells. In clinical trials, elotuzumab in combination with immunomodulatory drugs and proteasome inhibitors has demonstrated an excellent efficacy and safety profile in treating MM.

## Background

The paradigm of multiple myeloma (MM) therapy has changed dramatically in the past two decades. Immunomodulatory drugs (IMiDs) including thalidomide and lenalidomide and proteasome inhibitors (PIs) such as bortezomib and carfilzomib emerged as novel agents with high efficacy [1–3] and greatly improved overall survival (OS) of patients with MM [4]. Despite these significant advances, most patients still relapse and eventually become treatment-resistant. The median OS of patients with disease double refractory to thalidomide/lenalidomide and bortezomib was only 9 months [5]. Recently, a plethora of new agents have emerged as effective therapies, including new generation of PIs (carfilzomib, ixazomib) and IMiD (pomalidomide), histone deacetylase inhibitors (panobinostat, vorinostat), and monoclonal antibodies (daratumumab, elotuzumab), among others, which will hopefully further improve MM treatment outcomes [6]. While the CD20 antibody rituximab plays a pivotal role in lymphoma treatment, efficacious monoclonal antibodies have been long awaited in MM. In November of 2015, the US Food and Drug Administration (FDA) approved the CD38 antibody daratumumab and SLAMF7 antibody elotuzumab for the treatment of MM. In this article, we review the development and mechanisms of action of elotuzumab and summarize available data from preclinical and clinical studies.

## SLAMF7 as a therapeutic target in MM

In an attempt to identify potential new therapeutic targets in MM, Hsi and colleagues [7] first took a subtractive hybridization approach to subtract naïve B cell complementary DNA (cDNA) from the memory B cell and plasma cell cDNA library. The remaining genes that were selectively expressed in plasma cells were screened for structural/functional classification and the potential for cell surface localization. SLAMF7 was one of the genes identified to be highly expressed in plasma cells and to encode a cell-surface protein against which antibodies could potentially be developed.

SLAMF7, also known as CS1 (CD2 subunit 1), belongs to the signaling lymphocytic activation molecule (SLAM) family of cell surface receptors that are important for immunomodulation [8, 9]. It was previously recognized as a NK cell surface receptor that was critical for NK cell activation [10–12]. Gene expression profiling revealed that SLAMF7 expression was restricted to leukocytes, primarily plasma cells, and NK cells, and absent in other normal tissues [7]. In addition, SLAMF7 was highly expressed in plasma cells from healthy donors, patients with monoclonal gammopathy of undetermined significance (MGUS), smoldering multiple myeloma (SMM), and MM regardless of molecular subtype [7].

Most SLAM family receptors, including SLAMF7, are self-ligands [8, 9]. Upon receptor engagement, the presence or absence of adaptor protein in the cell dictates stimulatory or inhibitory effect, respectively [8]. While most SLAM family receptors use SLAM-associated protein (SAP) and EWSFli1-activated transcript-2 (EAT-2) as adaptors, it appears that SLAMF7 only recruits EAT-2 [8, 9]. In NK cells, SLAMF7 interacts with EAT-2 and activates PI3K and PLC-γ signaling pathways, thereby exerts a positive effect on NK cell function [9, 12]. In the absence of EAT-2, SLAMF7 mediates an inhibitory effect [8]. Although plasma cells do not express EAT-2, SLAMF7 may utilize other mechanisms to promote myeloma cell growth and survival. Studies have shown that SLAMF7-mediated signaling is important for the interaction between myeloma cells and their adhesion to bone marrow stromal cells (BMSCs) and can activate ERK1/2, STAT3, and AKT pathways to promote survival [13, 14]. The expression profile, cell surface localization, and the cellular functions make SLAMF7 an excellent therapeutic target in MM.

## Elotuzumab development and preclinical studies in MM

Hsi and colleagues [7] initially developed two mouse monoclonal antibodies, MuLuc63 (IgG2a) and MuLuc90 (IgG2b), that recognize the extracellular domain of SLAMF7. Both antibodies exhibited in vivo anti-myeloma activity in a L363 xenograft model. Because MuLuc63 was significantly more potent, it was selected for humanization [15]. HuLuc63, the fully humanized version of MuLuc63, exhibited significant anti-tumor activity in L363, OPM2, and MM1S xenograft models [7, 13, 15]. This antibody was later named elotuzumab.

**Fig. 1:**
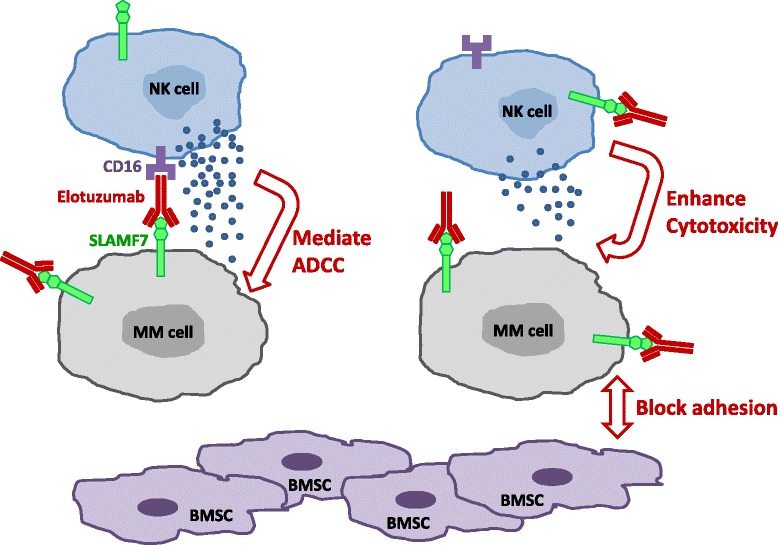
The mechanisms of action of elotuzumab. *Upper left*, elotuzumab binds SLAMF7 on MM cells, and its Fc fragment is then bound by CD16 on NK cells, mediating ADCC. *Upper right*, elotuzumab binds SLAMF7 on NK cells, directly enhancing its cytotoxicity. *Bottom right*, elotuzumab binds SLAMF7 on MM cells, inhibiting its interaction with BMSCs. *NK cell* natural killer cell; *MM cell* multiple myeloma cell; *BMSC* bone marrow stromal cell; *ADCC* antibody-dependent cellular cytotoxicity

The mechanisms of action of elotuzumab include mediating antibody-dependent cell-mediated cytotoxicity (ADCC) [7, 13], enhancing NK cell cytotoxicity [16], and disrupting MM cell adhesion to BMSC [13] (Fig. 1). In vitro ADCC studies showed that elotuzumab induced MM cell lysis by peripheral blood mononuclear cells (PBMCs) [13] and autologous or allogeneic NK cells [7]. Depletion of NK cells from PBMCs [13] or blocking the Fc receptor (CD16) on NK cells [7] significantly inhibited the ADCC activity. In the OPM2 xenograft model, altering the HuLuc63 affinity with CD16 affected its anti-myeloma activity accordingly, and depletion or suppressing the function of host NK cells also abolished its anti-myeloma activity. These studies suggest that NK cells are the dominant effectors that mediate elotuzumab-induced ADCC against MM cells. Collins et al. demonstrated that elotuzumab can also bind SLAMF7 on NK cells and enhance its cytotoxicity towards MM cells in an ADCC-independent manner [16]. Elotuzumab, as well as its G2M3 variant with defective CD16 binding, increased CD69 expression, IFN-γ production, and granzyme B (GrB) secretion of NK cells, suggesting that elotuzumab can activate NK cells through SLAMF7 binding. Elotuzumab facilitated SLAMF7 interaction with EAT-2 and enhanced phosphorylation of ERK in NK cells, confirming a direct role of elotuzumab on NK cell activation through SLAMF7 ligation [16]. Interestingly, elotuzumab and its F(ab’)_2_ fragment can stabilize the interaction between SLAMF7 on NK cells and MM cells and enhance MM cell killing, again suggesting the presence of elotuzumab-induced ADCC-independent cytotoxicity of NK cells against MM cells [16]. Furthermore, Tai et al. showed that elotuzumab can inhibit MM cell adhesion to BMSCs and may overcome the stimulatory effects of BMSCs on MM growth and survival in co-culture experiments [13], indicating that targeting myeloma-microenvironment interaction is an additional anti-myeloma mechanism of elotuzumab as summarized above.

While initial xenograft studies have demonstrated significant anti-myeloma activity of elotuzumab [7, 13], combination of elotuzumab with other agents appeared to have superior activities. Van Rhee and colleagues showed that elotuzumab in combination with bortezomib had better anti-tumor activity than either single agent alone in an OPM2 xenograft model [17]. Similarly, Balasa et al. demonstrated that adding lenalidomide enhanced the anti-myeloma activity of elotuzumab in the OPM2 xenograft model [18]. These studies provided the rationale for testing elotuzumab-containing combinational therapy for MM in clinical trials. Modulating NK cell-mediated ADCC by activating the CD137 receptor with an agonistic antibody or blocking killer immunoglobulin-like receptor (KIR)-mediated inhibitory signaling with lirilumab has been shown to enhance the anti-myeloma activity of elotuzumab [19, 20]. Preliminary ex vivo studies have shown that elotuzumab can enhance the cytotoxicity of ex vivo expanded NK (ENK) cells towards MM cells [21, 22]. These combinational immunotherapies warrant further investigation.

## Elotuzumab for MM treatment in clinical trials

Clinical trials of elotuzumab that have data reported are summarized in Table 1. The first-in-human study of elotuzumab was a phase 1 trial evaluating its safety, tolerability, pharmacokinetics, and pharmacodynamics [23]. Thirty-five patients with MM who had received at least two lines of therapy were enrolled, with a median age of 64.5 years and a median of 4.5 lines of therapy. Using a standard 3 + 3 dose escalation approach, 34 patients were treated, with doses ranging from 0.5 to 20 mg/kg. The maximum tolerated dose (MTD) was not reached with dose escalation up to the maximum planned dose (MPD). Common treatment-related adverse events (AEs) included chills, fatigue, pyrexia, cough, headache, anemia, nausea, and back pain, most of which were grade 1–2. Only two grade 3–4 AEs were attributed to elotuzumab, including one grade 3 hypersensitivity and one grade 4 acute renal failure. Transfusion reactions appeared common although most were grade 1–2, necessitating a protocol amendment to include premedication before first infusion. Commonly seen AE with other anti-myeloma agents such as peripheral neuropathy with bortezomib and thalidomide were not observed with elotuzumab [24, 25]. SLAMF7 on bone marrow-derived MM cells was saturated at doses of 10 and 20 mg/kg. After a median of four infusions at 2-week intervals, nine patients had stable disease (SD), but no objective response was observed. However, it should be noted that only three patients were treated at the dose that has been FDA approved (10 mg/kg).

**Table 1:** Major clinical trials of elotuzumab that have published data

Study	NCT number (trial name)	Phase	*N*	Regimen	ORR	PFS rate	Median PFS (months)
Zonder 2012 [23]	NCT00425347 (HuLuc63-1701)	1	35^a^	Elotuzumab (0.5–20 mg/kg)	0	–	–
Lonial 2012 [26, 27]	NCT00742560 (HuLuc63-1703)	1b	29^b^	Elotuzumab (5–20 mg/kg) + Rd	82 %	–	32.9
Richardson 2015 [27]	NCT00742560 (HuLuc63-1703)	2	36	Elotuzumab (10 mg/kg) + Rd	92 %		32.5^c^
			37	Elotuzumab (20 mg/kg) + Rd	76 %		25.0^c^
Lonial 2015 [32]	NCT01239797 (ELOQUENT-2)	3	321	Elotuzumab (10 mg/kg) + Rd	79 %	1-year 68 %, 2-year 41 %	19.4
			325	Rd	66 %	1-year 57 %, 2-year 27 %	14.9
Mateos 2014 [34]	NCT01632150	2	40	Elotuzumab (10 mg/kg) + TD^d^	40 %	–	–
Jakubowiak 2012 [35]	NCT00726869 (HuLuc63-1702)	1	28	Elotuzumab (2.5–20 mg/kg) + V	48 %		9.46^e^
Palumbo 2015 [38] Jakubowiak 2016 [39]	NCT01478048	1/2	77^f^	Elotuzumab (10 mg/kg) + VD	66 %	1-year 39 %, 2-year 18 %	9.7
			75	VD	63 %	1-year 33 %, 2-year 11 %	6.9

*N* number; *ORR* objective response rate; *PFS* progression-free survival; *Rd* lenolidomide and dexamethasone; *TD* thalidomide and dexamethasone; *V* bortezomib; *VD* bortezomib and dexamethasone

^a^Thirty-four patients treated

^b^Twenty-eight patients treated

^c^Median time to progression or death

^d^Cyclophosphamide was added if progressing between cycle 2 and 5 or not responding by cycle 5

^e^Median time to progression

^f^Seventy-five patients treated

Subsequent clinical studies have focused on elotuzumab in combination with other agents such as IMiDs and PIs. In the phase 1b part of the 1703 study, elotuzumab at escalating doses (5, 10, and 20 mg/kg) was combined with lenalidomide and dexamethasone (Rd) for treatment of relapsed or refractory (RR) MM [26]. Twenty-nine patients were enrolled, with a median age of 60 and a median of 3 prior therapies. No dose-limiting toxicity (DLT) was observed during dose escalation, and the 20 mg/kg cohort was expanded. Common AEs included fatigue, anemia, diarrhea, nausea, constipation, and neutropenia; most frequently reported grade 3–4 AEs were neutropenia (36 %) and thrombocytopenia (21 %). Infusion reactions were observed in 89 % of the patients, most of which were grade 1–2. In the updated report, objective response rate (ORR) was 82 % for the 28 treated patients, with 1 (4 %) complete response (CR), 12 (43 %) very good partial response (VGPR), and 10 (36 %) partial response (PR) [27]. For the 20 mg/kg cohort (22 patients, treated until disease progression), median duration of response (DOR) has not been reached after a median follow-up of 16.4 months [26]. According to the updated report, the median progression-free survival (PFS) was 32.9 months overall [27].

The phase 2 part of the 1703 study evaluated elotuzumab (10 or 20 mg/kg through randomization) in combination with Rd for RR MM in 73 patients [27]. The median age was 62, and patients had received 1–3 prior lines of therapy. The median follow-up was 21.2 months for the 10 mg/kg group (36 patients) and 16.8 months for the 20 mg/kg group (37 patients). The overall ORR was 84 %, with 3 (4 %) stringent CR, 7 (10 %) CR, 31 (42 %) VGPR and 20 (27 %) PR. The ORR was 92 and 76 % for the 10 and 20 mg/kg groups, respectively. The median time to initial/best response was 1.0/2.8 months for the 10 mg/kg group and 1.7/2.4 months for the 20 mg/kg group. The median DOR was 34.8 and 29 months for the 10 and 20 mg/kg groups, respectively. The median time to progression (TTP) or death was 32.5 and 25.0 months for the 10 and 20 mg/kg groups, respectively, and 28.6 months overall. The safety profiles were similar between the two groups. The most common grade 3–4 AE was cytopenia. Implementation of a premedication regimen significantly reduced infusion reaction occurrence to 11 % of the patients (mostly grade 1–2).

Compared with historical data on Rd [28–31], the 1703 study demonstrated that addition of elotuzumab improved the ORR (84 vs. 48–67 %) and PFS (28.6 vs. 11–18 months) in patients with RR MM [27]. The ELOQUENT-2 study directly compared the efficacy of elotuzumab plus Rd versus Rd alone for RR MM [32]. Based on results of the 1703 study, the dose of elotuzumab in this phase 3 randomized trial was 10 mg/kg. In total, 646 patients were enrolled, with a median age of 66 and a median of 2 prior therapies. Seventy percent of patients had received bortezomib before, and 48 and 6 % of patients had prior exposure to thalidomide and lenalidomide, respectively. About one third of patients had 17p deletion. The median follow-up was 24.5 months. The ORR was 79 % for the elotuzumab group (321 patients) and 66 % for the control group (325 patients) (odds ratio 1.9). The PFS rate was 68 vs. 57 % for the elotuzumab and control groups at 1 year and 41 vs. 27 % at 2 years. The median PFS was 19.4 vs. 14.9 months for the elotuzumab and control groups (hazard ratio 0.70). The ORR and PFS in the elotuzumab group appeared inferior to those in the 1703 study, but it should be noted that patients in this trial were older, and more patients had high-risk cytogenetic profiles and coexisting illnesses [27, 32]. Cytopenias were the most common grade 3–4 AEs and occurred at similar rates in both groups, except for a higher rate of lymphocytopenia in the elotuzumab group (77 vs. 49 %), which was likely due to lymphocyte trafficking. With mandatory premedication, infusion reaction occurrence was 10 % in the elotuzumab group. Results of the ELOQUENT-2 study clearly proved the benefit of adding elotuzumab to Rd for the treatment of RR MM. Based on these data, FDA approved elotuzumab to be used in combination with lenalidomide and dexamethasone for MM patients who had received one to three prior therapies.

The ELOQUENT-1 trial, which studies Rd with or without elotuzumab for untreated MM, is currently ongoing. A recent phase 1b trial studied the pharmacokinetics and safety of the elotuzumab plus Rd combination in 26 patients with various renal function and demonstrated that patients with severe renal impairment (creatinine clearance [CrCl] < 30 mL/min, not requiring dialysis) and end-stage renal disease (requiring dialysis) had identical elotuzumab pharmacokinetics and safety profile compared with patients with normal renal function (CrCl > 90 mL/min), suggesting that elotuzumab does not require dose adjustment in MM patients with renal dysfunction [33]. The combination of elotuzumab with another IMiD, thalidomide, was studied in a phase 2 trial [34]. Forty patients were enrolled, with a median age of 64 and a median of 3 prior therapies. Patients received elotuzumab in combination with thalidomide and dexamethasone (TD) and cyclophosphamide if progressing between cycle 2 and 5 or not responding by cycle 5. Except for infusion reactions, elotuzumab caused minimal incremental toxicity when added to TD. The ORR was 40 %, and 63 % of patients maintained response at 1 year.

The combination of elotuzumab and PIs has also been studied. A phase 1 trial studied the elotuzumab and bortezomib combination for patients with RR MM [35]. Twenty-eight patients with a median age of 63 and a median of 2 prior therapies were enrolled. During elotuzumab dose escalation (2.5 to 20 mg/kg), no DLT was observed and MTD was not reached up to MPD. The most frequent grade 3–4 AEs were lymphopenia and fatigue. In 27 evaluable patients, the ORR was 48 %. The median TTP was 9.46 months. Compared with previous data on bortezomib monotherapy for RR MM [3, 36, 37], the elotuzumab plus bortezomib combination appeared to have better ORR (48 vs. 27-41 %) and TTP (9.46 vs. 6.22–7 months). A phase 2 randomized trial compared elotuzumab in combination with bortezomib and dexamethasone (Vel/Dex) vs. Vel/Dex alone for patients with RR MM [38, 39]. In total, 152 patients were randomized, with a median age of 66. The ORR was 66 % in the elotuzumab arm (77 patients) and 63 % in the control arm (75 patients). The 1-year PFS rate was 39 and 33 %, and 2-year PFS rate was 18 and 11 %, for the elotuzumab and control arm, respectively. The median PFS was 9.7 months in the elotuzumab arm and 6.9 months in the control arm. The PFS rates were lower and the PFS was shorter than those seen in the ELOQUENT-2 study in which Rd was used [32]. Nevertheless, data from this phase 2 study suggest that addition of elotuzumab to bortezomib-based therapy may provide additional benefit for patients with RR MM.

Adding elotuzumab to the IMiD/PI combination is also under investigation. The SWOG study S1211 was designed to evaluate the efficacy of adding elotuzumab to lenalidomide, bortezomib, and dexamethasone (RVd) for the front line therapy of high risk MM. In the phase 1 portion, newly diagnosed symptomatic MM patients regardless of risk were eligible. Eight patients were enrolled and six received treatment. At the time of report, all six patients had completed 8 cycles of induction therapy with elotuzumab in combination with RVd, and five had completed at least 4 cycles of maintenance therapy with dose-attenuated RVd and elotuzumab. The phase 1 safety data demonstrated that elotuzumab did not lead to major additive AEs beyond those known for RVd [40]. The phase 2 part of the trial is ongoing and will provide the efficacy data. Some additional ongoing trials of elotuzumab in combination with IMiDs, PIs and/or other novel agents are listed in Table 2.

**Table 2:** Major ongoing clinical trials of elotuzumab

NCT number	Title	Ph	*N*	Recruitment
NCT01335399 (ELOQUENT-1)	Phase III study of lenalidomide and dexamethasone with or without elotuzumab to treat newly diagnosed, previously untreated multiple myeloma	3	750	Active, not recruiting
NCT02272803	Phase II study of lenalidomide/dexamethasone with or without elotuzumab for newly diagnosed MM patients in Japan	2	80	Recruiting
NCT01241292	Japanese study of BMS-901608 (Elotuzumab) in combination with lenalidomide and low dose dexamethasone	1	7	Active, not recruiting
NCT02159365	Study of safety of elotuzumab administered over approximately 60 min in combination with lenalidomide and dexamethasone for newly diagnosed or relapsed/refractory multiple myeloma patients	2	76	Active, not recruiting
NCT01393964	Study of elotuzumab in combination with lenalidomide and dexamethasone in subjects with multiple myeloma and various levels of renal function	1	35	Active, not recruiting
NCT02279394	Trial of combination of elotuzumab and lenalidomide +/− dexamethasone in high-risk smoldering multiple myeloma	2	82	Recruiting
NCT02420860	Study of elotuzumab with lenalidomide as maintenance after autologous stem cell transplant (ASCT)	2	100	Recruiting
NCT02655458	Elotuzumab in autologous stem cell transplantation (ASCT) and lenalidomide maintenance for multiple myeloma	1	15	Recruiting
NCT02612779	A study of elotuzumab in combination with pomalidomide and low dose dexamethasone (EPd) in patients with multiple myeloma relapsed or refractory to prior treatment with lenalidomide	2	60	Recruiting
NCT02654132	Trial of pomalidomide and low-dose dexamethasone with or without elotuzumab to treat refractory and relapsed and refractory multiple myeloma (ELOQUENT-3)	2	121	Recruiting
NCT01668719 (S1211)	Bortezomib, dexamethasone, and lenalidomide with or without elotuzumab in treating patients with newly diagnosed high-risk multiple myeloma	1/2	122	Recruiting
NCT02375555	Study of bortezomib, lenalidomide, dexamethasone & elotuzumab in newly diagnosed MM	2	40	Recruiting
NCT02495922 (GMMG HD6)	A phase III trial on the effect of elotuzumab in VRD induction/consolidation and lenalidomide maintenance in patients with newly diagnosed myeloma	3	516	Recruiting
NCT02718833	A study of elotuzumab with pomalidomide, bortezomib, and dexamethasone in relapsed multiple myeloma	2	46	Recruiting
NCT02726581	Study of combinations of nivolumab, elotuzumab, pomalidomide and dexamethasone in multiple myeloma	3	406	Recruiting
NCT02252263	A phase I open label study of the safety and tolerability of elotuzumab (BMS-901608) administered in combination with either lirilumab (BMS-986015) or urelumab (BMS-663513) in subjects with multiple myeloma	1	136	Active, not recruiting

*Ph* phase; *N* number

## Conclusions

Elotuzumab has demonstrated excellent efficacy and safety profiles when combined with an IMiD or PI for the treatment of RR MM. There was only limited data on single agent elotuzumab for RR MM, with only three patients treated at the FDA approved dose in the initial phase 1 study [23]. Further investigations should be done to evaluate its efficacy when used alone. Ongoing clinical trials will further define the efficacy of elotuzumab with IMiD or PI combinations in both untreated and RR MM patients and evaluate the four drug combination (elotuzumab, IMiD, PI, and corticosteroid) for MM treatment. Importantly, so far, there has been no data demonstrating the efficacy of elotuzumab in lenalidomide-refractory patients. How to overcome this significant limitation requires further investigation. The use of elotuzumab as part of induction and/or maintenance therapy is under investigation. Whether combining elotuzumab with chemotherapy is beneficial in MM needs to be studied. Combining elotuzumab with daratumumab is worth investigating. Hitting CD38 and SLAMF7 simultaneously may provide a higher MM cell killing efficacy and may overcome potential resistance to either agent. Daratumumab reduces NK cell levels, and perhaps combining daratumumab with elotuzumab may be effective in overcoming this effect. Combining elotuzumab with other immunotherapies such as anti-PD1 antibodies, anti-KIR antibodies, and CD137 agonizing antibodies are under investigation in clinical trials already. Anti-PD1 therapies may activate T cell-mediated MM cell killing mechanisms, adding to the NK cell-mediated anti-myeloma activities. Activating the CD137 receptor or blocking the KIR-mediated inhibitory signaling can modulate NK cell-mediated ADCC, and combining elotuzumab with these agents may lead to enhanced MM cell killing by ADCC [19, 20]. With expanding options available, when and how to use each agent will be an ever-evolving question. Related biomarkers on response and prognosis are to be studied. Whether elotuzumab, alone or in combination with other regimens, provides a different efficacy and safety profile in a specific population requires further study [30, 41].

## Abbreviations

ADCC, antibody dependent cell-mediated cytotoxicity; AE, adverse event; BMSC, bone marrow stromal cells; CR, complete response; DLT, dose-limiting toxicity; DOR, duration of response; IMiD, immunomodulatory drug; MM, multiple myeloma; MPD, maximum planned dose; MTD, maximum tolerated dose; NK, natural killer; ORR, objective response rate; OS, overall survival; PBMC, peripheral blood mononuclear cell; PFS, progression-free survival; PI, proteasome inhibitors; PR, partial response; Rd, lenalidomide and dexamethasone; RR, relapsed or refractory; RVd, lenalidomide, bortezomib and dexamethasone; SD, stable disease; TD, thalidomide and dexamethasone; TTP, time to progression; Vel/Dex, bortezomib and dexamethasone; VGPR, very good partial response
